# A correlation propagation model for nonlinear fourier transform of second order solitons

**DOI:** 10.1038/s41598-021-82011-y

**Published:** 2021-01-28

**Authors:** Wen Qi Zhang, Terence H. Chan, V. Shahraam Afshar

**Affiliations:** 1 Institute for Telecommunications Research, University of South Australia, Adelaide, Australia; 2 Laser Physics and Photonic Devices Laboratories, School of Engineering, University of South Australia, Adelaide, Australia

**Keywords:** Information technology, Nonlinear optics, Fibre optics and optical communications

## Abstract

Inverse scattering transform or nonlinear Fourier transform (NFT) has been proposed for optic communication to increase channel capacity beyond the well known Shannon limit. Within NFT, solitons, as discrete outputs of the transform, can be a type of resource to carry information. Second-order solitons as the most basic higher order solitons show correlations among their parameters in the nonlinear Fourier domain as they propagate along a fibre. In this work, we report, for the first time, a correlation propagation model for second-order soliton pulses in the nonlinear Fourier domain. The model can predict covariance matrices of soliton pulses at any propagation distance using only the covariance matrices calculated at the input of the fibre with different phases in the nonlinear Fourier domain without the need of propagating the pulses.

## Introduction

Fibre optic communications denote an indisputable impact on human everyday life with increasing demand for data transfer. Consequentially backbone data traffic is estimated to grow 45% per annum manifesting a difficult task for optical communication technologies to cope with such an immense capacity growth rate^1–3^. This is particularly difficult since fibre optic channels reach a fundamental maximum capacity, due to their nonlinear nature^2,3^, which is well known as the linear Shannon capacity^4^. Experiments have been able to approach this nonlinear-capacity-limit within a factor of two impeding further improvements and inevitably leading to what is known as a “Capacity Crunch”^1–3,5^.

Unlike traditional systems, originally designed for linear wireless and wired transmission channels, a novel approach widely known as the nonlinear Fourier transform (NFT) aspires to exploit fiber nonlinearity^5–7^. Under the NFT, a signal *q*(*t*) in time domain is transformed into a continuous, $Q^c(\lambda )$, and a discrete, $Q^d(\lambda _k)$, spectral part, with continuous and discrete eigenvalues $\lambda$, and $\lambda _k$, respectively^8^.

This special signal family denotes a certain immunity against nonlinear effects and fibre dispersion, where nonlinear eigenvalues remain invariant and spectral magnitudes acquire linear complex phases, see Eq. (7), as the signal propagates through an optical fibre^5^. Given these attributes it is not surprising that eigenvalues pose as desirable information carriers which is demonstrated by recent studies^9–16^. These studies have proposed the choice of either eigenvalue-pairs, or the spectral phases to encode data. In the discrete NFT domain, encoding data on the phase of the NFT spectral components $Q^d(\lambda _k)$ is mainly through QPSK modulation^9–11,13^.

The invariance of nonlinear eigenvalues and simple linear phase change of spectral magnitudes of optical signals through fibre propagation in NFT domain, Eq. (7), are only valid for noiseless transmission. In practice, noise can be generated in transmitters and receivers, and also during propagation through a fibre. Hence, to implement or improve NFT-based encoding/decoding schemes, it is crucial to have the knowledge of noise perturbation on spectral phases, amplitudes and eigenvalues, which eventually determines the channel capacity^4^. Focusing on signals with discrete eigenvalues (soliton signals), correlations among the noise perturbation of eigenvalues and correlation between eigenvalues and their corresponding phases have recently been observed and reported^9,16,17^. Periodic patterns as functions of NFT phase difference were observed in the correlations between eigenvalues^16^ and phases in a back to back system (no signal propagation)^18^. These results were confirmed in the work^17^ which was developed independently at the same time. The work in^17^ also implies the periodic behaviour in the correlations between $|Q^d_k|$. However, there is not yet a noise model that can predict correlations among NFT parameters of a multi-soliton signal as a function of propagation length.

In this report, we focus on building a correlation propagation model for second order soliton signals (two eigenvalues) that includes all degrees of freedom. The model assumes ideal Raman amplification supporting a constant signal power throughout the length of the fibre. The study of correlation properties when there is significant power loss in the signal is not in the scope of this work.

The main body of this report is structured as following: we first introduce a few fundamental equations of NFT that are relevant to our discussion, then we present our correlation propagation model and discuss the parameter space of second-order soliton pluses. Finally we conclude our work.

### Nonlinear Fourier transform

The evolution of a noisy signal inside a lossless optical fibre in the nonlinear regime can often be expressed using the following stochastic nonlinear Schrödinger equation

1 $$\begin{aligned} \frac{\partial }{\partial z}q(t,z) = -j\frac{\partial ^2}{\partial t^2}q(t,z) - 2j\left| q(t,z)\right| ^2 q(t,z)+j\kappa N(t,z) \end{aligned}.$$

where $\kappa$ is the noise strength, and *N*(*t*, *z*) is a Gaussian white noise term that follows the rule

2 $$\begin{aligned} \mathrm {E}\left[ N(t,z)N^{*}(t^{\prime },z^{\prime })\right] =\delta (t-t^{\prime })\delta (z-z^{\prime }). \end{aligned}$$

When noise is zero ($\kappa =0$), Eq. (1) can be solved analytically using the inverse scattering method^19^, also called Nonlinear Fourier Transform (NFT), where a corresponding scattering problem associated with Eq. (1) can be found as

3 $$\begin{aligned} \frac{\partial }{\partial t}\left( \begin{array}{l} v_1(t,z) \\ v_2(t,z) \end{array} \right) =\left( \begin{array}[c]{ccc} - i\lambda &{} q(t) &{} \\ -q^{*}(t) &{} i\lambda &{} \end{array} \right) \left( \begin{array}{l} v_1(t,z) \\ v_2(t,z) \end{array} \right) \text {,} \end{aligned}$$

with the boundary condition

4 $$\begin{aligned} \left( \begin{array}{l} v_1(t,z) \\ v_2(t,z) \end{array} \right) = \left( \begin{array}{l} 1 \\ 0 \end{array} \right) e^{-j\lambda t},&\quad t\rightarrow -\infty \text {.} \end{aligned}$$

Two scattering coefficients can be defined as

5 $$\begin{aligned} a(\lambda ,z)&=\lim _{t\rightarrow +\infty }v_1(t,z)e^{j\lambda t}\text {,} \end{aligned}$$

6 $$\begin{aligned} b(\lambda ,z)&=\lim _{t\rightarrow +\infty }v_2(t,z)e^{-j\lambda t}\text {.} \end{aligned}$$

The NFT is defined as a transformation from $q(t,z) \rightarrow Q^c(\lambda ,z)$ and $Q^d(\lambda _k,z)$, where the continuous part of the spectrum $Q^c(\lambda ,z)= \frac{b(\lambda )}{a(\lambda )}$ for real $\lambda$ and the discrete part of the spectrum $Q^d(\lambda _k,z)=\frac{b(\lambda )}{a^\prime (\lambda )}$ for discrete points of $\lambda _k$ in the upper complex plane that is corresponding to the roots of $a(\lambda )$. In the nonlinear Fourier domain, the propagation of the nonlinear spectra of a pulse can be expressed as:

7a $$\begin{aligned} Q^{c}(\lambda ,z)&= |Q^{c}(\lambda ,0)|e^{j[\angle Q^{c}(\lambda ,0) -4\lambda ^2 z]} \text { ,} \end{aligned}$$

7b $$\begin{aligned} Q^{d}(\lambda _k,z)&= |Q^{d}(\lambda _k,0)|e^{j[\angle Q^{d}(\lambda _k,0)-4\lambda _k^2 z]} \text { ,} \end{aligned}$$

where $|Q^c(\lambda ,0)|$ and $|Q^d(\lambda _k,0)|$ are the initial NFT spectral magnitude and $\angle Q^c(\lambda ,0)$ and $\angle Q^d(\lambda _k,0)$ are the initial phase at the beginning of the fibre. In this work, we focus on the discrete part of the spectrum and use subscripts $|Q^d_k|$ and $\angle Q^d_k$ to represent the discrete spectral magnitude and phase associated with the discrete eigenvalue $\lambda _k$. Using Eq. (7b), one can find the NFT spectral magnitude and phase at position *z* as,

8a $$\begin{aligned} |Q^d_k(z)|&= |Q^{d}(\lambda _k,0)|e^{4\text {Im}(\lambda _k^2)z} \text { ,} \end{aligned}$$

8b $$\begin{aligned} \angle Q^d_k(z)&= \angle Q^d_k(0)-4\text {Re}(\lambda _k^2)z \text { .} \end{aligned}$$

Considering a pulse with two eigenvalues $\lambda _1$ and $\lambda _2$ propagating along a fibre, due to the differences in $\lambda _1$ and $\lambda _2$, $\angle Q^d_1$ and $\angle Q^d_2$ increase at a different rate as the propagation distance increase. We define the difference between the two phases as the “*NFT phase difference*” $\Phi$, where

9 $$\begin{aligned} \Phi (z) = \angle Q^d_1(0)-\angle Q^d_2(0)-4z Re(\lambda ^2_1-\lambda ^2_2) \text {.} \end{aligned}$$

Previously, we discovered that the correlation between eigenvalues in multi-eigenvalue systems is influenced by the NFT phase difference due to propagation, i.e., $4z Re(\lambda ^2_1-\lambda ^2_2)$^16^. In general, Eqs. (8) indicate that in the NFT domain, propagating a pulse noiselessly and losslessly is equivalent to scale its spectral magnitude and change its initial phase.

### Correlation propagation model

Optical solitons are pulses with only discrete eigenvalues. In this paper, we investigate the “correlation” between eigenvalues and the spectral magnitudes or phases associated with the eigenvalues for two-eigenvalue solitons (also as known as second order solitons).

Previous work^16^ shows that the correlation of the imaginary part of the eigenvalues of a collection of 2*sech*(*t*) soliton pulses change periodically as the pulse propagates along a fibre. In general, both eigenvalues and associated $Q^d(\lambda _k)$ shall be perturbed by noise. Hence, a covariance matrix $\Lambda$ associated to the perturbed eigenvalues and spectra of a second order soliton signal can be written as an 8 by 8 matrix, in which

10 $$\begin{aligned} \Big [\Lambda \Big (Re(\lambda _1),Re(\lambda _2),Im(\lambda _1),Im(\lambda _2), |Q^d_1|,|Q^d_2|,\angle Q^d_1,\angle Q^d_2\Big )\Big ]_{ij}=\sigma _{i,j} . \end{aligned}$$

where $i,j\in \big [Re(\lambda _1),Re(\lambda _2),Im(\lambda _1),Im(\lambda _2), |Q^d_1|,|Q^d_2|,\angle Q^d_1,\angle Q^d_2\big ]$, $\sigma _{i,j}$ for $i \ne j$ is the covariance of *i* and *j*, $\sigma _{i,i}=\sigma ^2_i$ is the variance of *i* and $\sigma _i$ is the standard deviation of *i*. It is worth mentioning that the covariance matrix is defined with respect to a given *q*(*t*) with the addition of a white Gaussian noise.

To quantify the correlation, we consider the correlation coefficient

11 $$\begin{aligned} \rho _{i,j} = \frac{\sigma _{i,j}}{\sigma _i\sigma _j}\text { ,} \end{aligned}$$

and to simplify our notation, a two-eigenvalue pulse will be denoted in time and NFT domain, respectively, by $q(t) \Leftrightarrow (Q_k^d, \lambda _k, \, k=1,2)$.

### Propagation and noise addition

Previous work^16^ suggests that there are different types of perturbations from different sources, .i.e, transmitter, amplifier or receiver. The perturbation model for eigenvalues, which was developed in the previous work^16^, led to two main conclusions: (1) the overall perturbation in eigenvalues can be approximated as the linear addition of all the perturbation from different sources and (2) the distributed noise along a fibre can be approximated by a set of point noise. Here, we review the main points of the model and expand it to include spectral magnitudes and phases.

**Figure 1:**
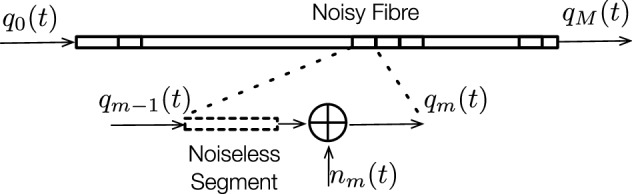
Noise addition model. Fibre is divided into “*M*” segments. In each segment, the noise is modelled by approximating it with a noiseless propagation and point noise added at the end of the propagation. The perturbations of eigenvalues and spectra between segments are assumed to be independent. The total perturbation at the end of the fibre is approximated as a linear addition of the perturbations in each segment plus a compensations of the spectral phase based on the distances from each segment to the end of the fibre.

A noisy fibre of length *L* is divided into *M* equally separated segments as shown in Fig. 1. Within each segment, the noise distributed throughout the segment is approximated by a single point noise $n_m(t)$ added at the end of the segment, while the segment itself becomes noiseless. A soliton pulse $q_m(t)$ propagates through the segment *m* of the fibre noiselessly. The perturbation in eigenvalues and $Q^d_k$ within the segment is then evaluated using the pulse $q^\prime _m(t)=q_m(t)+n_m(t)$.

The influence of noise of the previous segment has negligible effects on the current segment^16^. The perturbations between segments are considered independently. Hence, the total perturbation in $\lambda$ and $Q^d_k$ at the end of the fibre can be approximated as the sum of the perturbation in each segment. Any other point noise sources, i.e. transmitter noise and receiver noise, can be added to the corresponding segments where the noise sources sit. As a consequence, the correlation matrix of the 8 degrees of freedom of a two-soliton pulse at the end of the propagation can be written as the summation of the correlation matrices that only have noise in one of the segments along the fibre. In other words,

12 $$\begin{aligned}&\Lambda \Big (Re(\lambda _1(L)),Re(\lambda _2(L)),Im(\lambda _1(L)),Im(\lambda _2(L)), |Q^d_1(L)|,|Q^d_2(L)|,\angle Q^d_1(L),\angle Q^d_2(L)\Big )\nonumber \\&\quad =\sum _{m=1}^M \Lambda \Big (Re(\lambda _{1m}(L)),Re(\lambda _{2m}(L)),Im(\lambda _{1m}(L)), Im(\lambda _{2m}(L)),|Q^d_{1m}(L)|,|Q^d_{2m}(L)|,\angle Q^d_{1m}(L),\angle Q^d_{2m}(L)\Big )\text {,} \end{aligned}$$

where the subscript *m* denotes the segment in which the noise is added.

Since the eigenvalues remain unchanged during noiseless and lossless propagation, $\lambda _{km}(L)$ can be replaced by $\lambda _{km}(z_m)$ where $z_m$ is the distance from the input of the fibre to the end of segment *m*. For spectral amplitude $Q^d_k$, its changed is determined by Eq. (7b) for noiseless and lossless propagation. Hence, $Q^d_{km}(L)$ can be replaced by $Q^d_{km}(z_m) e^{-4j \lambda _{km}^2(z_m) l_m}$, where $l_m=L-z_m$. Eq. (12) can be rewritten as

13 $$\begin{aligned}&\Lambda \Big (Re(\lambda _1(L)),Re(\lambda _2(L)),Im(\lambda _1(L)),Im(\lambda _2(L)), |Q^d_1(L)|,|Q^d_2(L)|,\angle Q^d_1(L),\angle Q^d_2(L)\Big )\nonumber \\&\quad \sum _{m=1}^M \Lambda \Big (Re({\lambda _1}_m),Re({\lambda _2}_m),Im({\lambda _1}_m),Im({\lambda _2}_m), |{Q^d_1}_m e^{-4j{\lambda _1}^2_m l_m}|,|{Q^d_2}_m e^{-4j{\lambda _2}^2_m l_m}|,\angle {Q^d_1}_m e^{-4j{\lambda _1}^2_m l_m},\angle {Q^d_2}_m e^{-4j{\lambda _2}^2_m l_m}\Big )\text {,} \end{aligned}$$

where ‘$(z_m)$’ is suppressed to save space.

The model presented in Eq. (13) is inconvenient to computer. In order to calculate the covariance matrices on the RHS of the equation, one has to save all the instances of noisy $\lambda _k$ and $Q^d_k$ in all the segments and update them every time *L* changes. To simplify the model, the following approximation is applied to write the covariance matrices in the RHS of Eq. (13), which is evaluate at *L*, in terms of the covariance matrices evaluated at each segment ($L=z_m$).

By separating the real and imaginary part of $\lambda _k$ as $\lambda _k=Re(\lambda _k)+j Im(\lambda _k)$, we find

14 $$\begin{aligned} Q^d_k(z_m)e^{-4j\lambda ^2_{km} l_m}=Q^d_k(z_m)e^{8Re(\lambda _{km})Im(\lambda _{km})l_m}e^{-4j[Re(\lambda _{km})^2 -Im(\lambda _{km})^2]l_m}\text {.} \end{aligned}$$

Assume, the noiseless value for $\lambda _{km}$ is $\lambda _{k0}$, then we can write $\lambda _{km}$ as

15 $$\begin{aligned} \lambda _{km}=\lambda _{k0}+\Delta \lambda _{km} \end{aligned}$$

Replace $\lambda _{km}$ in Eq. (14) using Eq. (15) and assume $\Delta \lambda _{km}$ is sufficiently small, we get the following equations for the magnitude of the spectrum,

16 $$\begin{aligned}&|Q^d_k(z_m)e^{-4j\lambda ^2_{km}l_m}|=|Q^d_k(z_m)|e^{8Re(\lambda _{k0})Im (\lambda _{k0})l_m} e^{8[Re(\Delta \lambda _{km})Im(\lambda _{k0})+Re(\lambda _{k0}) Im(\Delta \lambda _{km})+Re(\Delta \lambda _{km})Im(\Delta \lambda _{km})]l_m} \nonumber \\&\quad \approx |Q^d_k(z_m)|e^{8Re(\lambda _{k0})Im(\lambda _{k0})l_m}+8|Q^d_k(z_m) |e^{8Re(\lambda _{k0})Im(\lambda _{k0})l_m}[Re(\Delta \lambda _{km})Im(\lambda _{k0}) +Re(\lambda _{k0})Im(\Delta \lambda _{km})]l_m \text {,} \end{aligned}$$

for the phase, no approximation is needed, hence

17 $$\begin{aligned} \angle \Big (Q^d_k(z_m)e^{-4j\lambda ^2_{km} l_m}\Big ) = \angle Q^d_k(z_m)e^{-4j[Re(\lambda _{km})^2-Im(\lambda _{km})^2]l_m} \text {.} \end{aligned}$$

We define $L_{km}=e^{8Re(\lambda _{k0})Im(\lambda _{k0})l_m}$, $A_k(z_m)=|Q^d_k(z_m)|[Re(\Delta \lambda _{km})Im(\lambda _{k0}) +Re(\lambda _{k0})Im(\Delta \lambda _{km})]$, $B_k(z_m)=Re(\lambda _{km})^2-Im(\lambda _{km})^2$, and the equations above can be rewritten as,

18a $$\begin{aligned} |Q^d_k(z_m)e^{-4j\lambda ^2_{km} l_m}|&\approx |Q^d_k(z_m)|L_{km} + 8 A_k(z_m)L_{km} l_m \text { ,} \end{aligned}$$

18b $$\begin{aligned} \angle \Big (Q^d_k(z_m)e^{-4j\lambda ^2_{km} l_m}\Big )&= \angle Q^d_k(z_m) - 4 B_k(z_m) l_m \text { .} \end{aligned}$$

Inserting Eqs. (18) into (13) and applying the covariance linear combination rules, each of the covariance matrices on the RHS of Eq. (13) can now be written as (subscript *m* is ignored for notation simplicity):

19 $$\begin{aligned}&\Lambda \Big (Re(\lambda _1),Re(\lambda _2),Im(\lambda _1),Im(\lambda _2),|Q^d_1 e^{-4j\lambda _1^2 l}|,|Q^d_2e^{-4j\lambda _2^2 l}|,\angle Q^d_1e^{-4j\lambda _1^2 l}, \angle Q^d_2e^{-4j\lambda _2^2 l}\Big )\nonumber \\&\quad ={\mathbb {L}}\odot \Big [\Lambda \Big (Re(\lambda _1),Re(\lambda _2),Im(\lambda _1), Im(\lambda _2),|Q^d_1|,|Q^d_2|,\angle Q^d_1,\angle Q^d_2\Big )\nonumber \\&\quad +{\mathbb {C}}\odot \Lambda \Big (Re(\lambda _1),Re(\lambda _2),Im(\lambda _1), Im(\lambda _2),A_1,A_2,B_1,B_2\Big )\Big ]+4l\cdot {\mathbb {L}}_0 \odot {\mathbb {U}}\text {,} \end{aligned}$$

where $\odot$ is the Hadamard product such that: $[A \odot B]_{ij}=A_{ij}B_{ij}$, and

20 $$\begin{aligned} {\mathbb {L}}= \begin{bmatrix} {\mathbb {O}} &{} {\mathbb {E}} \\ {\mathbb {E}}^T &{} {\mathbb {F}} \end{bmatrix}\text {,}&{\mathbb {L}}_0= \begin{bmatrix} {\mathbb {Z}} &{} {\mathbb {Z}} \\ {\mathbb {Z}} &{} {\mathbb {F}} \end{bmatrix}\text {,}&{\mathbb {C}}=4l\cdot \begin{bmatrix} {\mathbb {Z}} &{} {\mathbb {N}} \\ {\mathbb {N}}^T &{} 4l\cdot {\mathbb {M}} \end{bmatrix} \end{aligned}$$

21 $$\begin{aligned} {\mathbb {O}}= \begin{bmatrix} 1 &{} 1 &{} 1 &{} 1\\ 1 &{} 1 &{} 1 &{} 1\\ 1 &{} 1 &{} 1 &{} 1\\ 1 &{} 1 &{} 1 &{} 1 \end{bmatrix}\text {,}&{\mathbb {E}}= \begin{bmatrix} L_1 &{} L_2 &{} 1 &{} 1 \\ L_1 &{} L_2 &{} 1 &{} 1 \\ L_1 &{} L_2 &{} 1 &{} 1 \\ L_1 &{} L_2 &{} 1 &{} 1 \end{bmatrix}\text {,}&{\mathbb {F}}={\mathbb {E}}\odot {\mathbb {E}}^T\text {,} \end{aligned}$$

22 $$\begin{aligned} {\mathbb {Z}}= \begin{bmatrix} 0 &{} 0 &{} 0 &{} 0\\ 0 &{} 0 &{} 0 &{} 0\\ 0 &{} 0 &{} 0 &{} 0\\ 0 &{} 0 &{} 0 &{} 0 \end{bmatrix}\text {,}&{\mathbb {N}}= \begin{bmatrix} 2 &{} 2 &{} -1 &{} -1 \\ 2 &{} 2 &{} -1 &{} -1 \\ 2 &{} 2 &{} -1 &{} -1 \\ 2 &{} 2 &{} -1 &{} -1 \end{bmatrix}\text {,}&{\mathbb {M}}= \begin{bmatrix} 4 &{} 4 &{} -2 &{} -2 \\ 4 &{} 4 &{} -2 &{} -2 \\ -2 &{} -2 &{} 1 &{} 1 \\ -2 &{} -2 &{} 1 &{} 1 \end{bmatrix}\text {,} \end{aligned}$$

23 $$\begin{aligned} {\mathbb {U}}= \begin{bmatrix} {\mathbb {Z}} &{} {\mathbb {Z}} \\ {\mathbb {Z}} &{} {\mathbb {N}}\odot {\mathbb {V}}+{\mathbb {N}}^T\odot {\mathbb {V}}^T \end{bmatrix}\text {,}&{\mathbb {V}}= \begin{bmatrix} \sigma _{|Q^d_1|,A_1} &{} \sigma _{|Q^d_1|,A_2} &{} \sigma _{|Q^d_1|,B_1} &{} \sigma _{|Q^d_1|,B_2} \\ \sigma _{|Q^d_2|,A_1} &{} \sigma _{|Q^d_2|,A_2} &{} \sigma _{|Q^d_2|,B_1} &{} \sigma _{|Q^d_2|,B_2} \\ \sigma _{\angle Q^d_1,A_1} &{} \sigma _{\angle Q^d_1,A_2} &{} \sigma _{\angle Q^d_1,B_1} &{} \sigma _{\angle Q^d_1,B_2} \\ \sigma _{\angle Q^d_2,A_1} &{} \sigma _{\angle Q^d_2,A_2} &{} \sigma _{\angle Q^d_2,B_1} &{} \sigma _{\angle Q^d_2,B_2} \end{bmatrix}\text {.} \end{aligned}$$

The significance of Eq. (19) is that instead of saving all the instances of noisy $\lambda _k$ and $Q^d_k$, and re-evaluate them for every *L*, one only need to evaluate and save the three matrices on the RHS of Eq. (19) once for every segment. These matrices are independent of propagation length *L*. Inserting Eq. (19) into Eq. (13), we finally get

24 $$\begin{aligned}&\Lambda (Re(\lambda _1(L)),Re(\lambda _2(L)),Im(\lambda _1(L)),Im(\lambda _2(L)), |Q^d_1(L)|,|Q^d_2(L)|,\angle Q^d_1(L),\angle Q^d_2(L))\nonumber \\&\quad =\sum _{m=1}^M\Big \{ {\mathbb {L}}\odot \Big [\Lambda \Big (Re(\lambda _1),Re(\lambda _2), Im(\lambda _1),Im(\lambda _2),|Q^d_1|,|Q^d_2|,\angle Q^d_1,\angle Q^d_2\Big )\nonumber \\&\quad +{\mathbb {C}}\odot \Lambda \Big (Re(\lambda _1),Re(\lambda _2),Im(\lambda _1), Im(\lambda _2),A_1,A_2,B_1,B_2\Big )\Big ]+4l\cdot {\mathbb {L}}_0 \odot {\mathbb {U}} \Big \}\text {.} \end{aligned}$$

Since the signal propagates to each segment noiselessly, we can simply replace the propagation with adding a scaling factor and an extra phase to $Q^d_k$ according to Eqs. (8). To use the model, one needs to first calculate the covariance matrices for a few different $\Phi$ equally spaced within the range of [0,$2\pi$). The optimum number of points can be determined by gradually increasing the number of points and checking for convergence. One also need to keep in mind that $\Delta \lambda _{km}$ in all the segments need to satisfy the condition $8\text {Re}(\lambda _{k0}) \text {Im}(\Delta \lambda _{km}) l_m \ll 1$ or $8\text {Im}(\lambda _{k0}) \text {Re}(\Delta \lambda _{km}) l_m \ll 1$ in order for Eq. (16) to be valid. In this report, ten points is used. Once the covariance matrices for different $\Phi$ are obtained, map all the segments to corresponding $\Phi$ and scale the terms in the matrices associated with $|Q^d_k|$ by the factor $e^{4\text {Im}(\lambda _k^2)z_m}$, such as

$$\begin{aligned} \sigma _{|Q^d_k|e^{4\text {Im}(\lambda _k^2)z_m},x}=e^{4\text {Im}(\lambda _k^2)z_m} \sigma _{|Q^d_k|,x}\quad \text {and}\quad \sigma _{x,|Q^d_k|e^{4\text {Im}(\lambda _k^2)z_m}} =e^{4\text {Im}(\lambda _k^2)z_m}\sigma _{x,|Q^d_k|}\text {,} \end{aligned}$$

where *x* stands for any one of the parameters of the covariance matrices. Finally, use Eq. (24) to predict the covariance matrices at desired propagation distance.

With Eq. (24), one can predict the covariance matrix at any propagation distance by using a set of pre-calculated covariance matrices at the beginning of a fibre with different phases in $Q^d_k$ without the need of doing statistical pulse propagation calculations at all.

**Figure 2:**
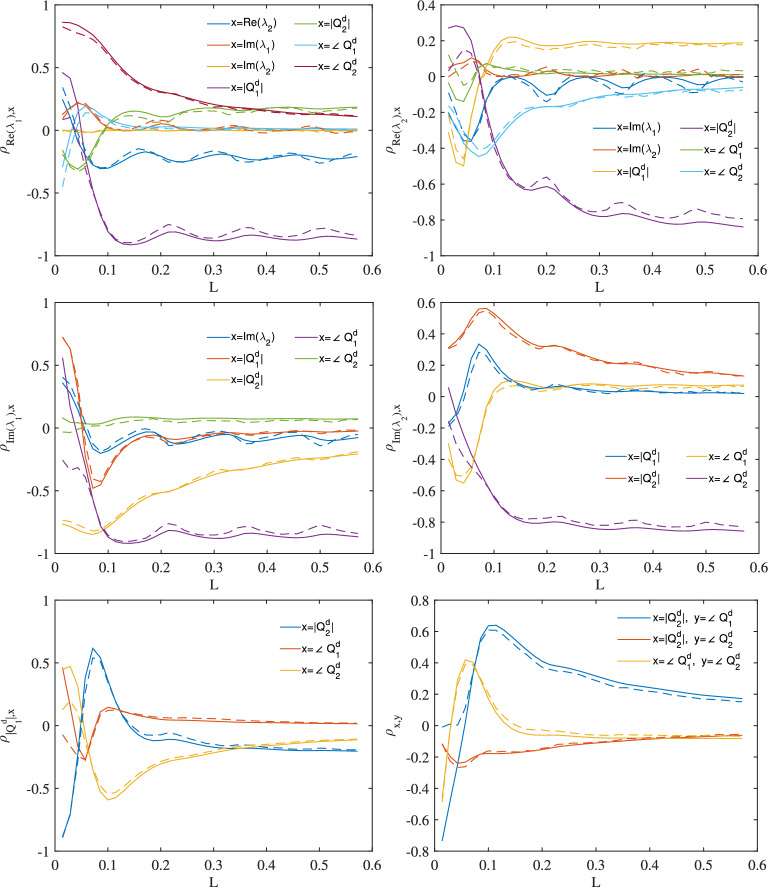
Noise linear addition approximation for the pulse with $\lambda _{1,2}=(6j,5j)$ and $Q^d_{1,2}=(-6j,-2j)$. Solid curves: model predictions, dashed curves: pulse propagation simulations.

Take a pulse with $\lambda _{1,2}=(6j,5j)$ and $Q^d_{1,2}=(-6j,-2j)$ as an example and consider a propagation distance $L_{2\pi }$ corresponding to $2\pi$ phase difference ($L_{2\pi }=2\pi /\left| 4Re(\lambda ^2_1-\lambda ^2_2)\right| \approx 0.143$ in this example), the corresponding correlation coefficient $\rho _{a,b}$ for all the top right half of the covariance matrix (symmetric with respect to the main diagonal axis) from $L=0$ to $4L_{2\pi }$ are plotted in Fig. 2. The values predicted using the model are plotted as solid curves and ones simulated through nonlinear pulse propagation are plotted as dashed curves. A Split-step Fourier method is applied for the pulse propagation simulation^20^ and the rest of the simulation details can be found in the section “Numerical settings”. As one can see, the model can produce reasonably good predictions of the covariance matrix at any propagation distance. Note that the length is a normalised unitless length. It can be scale to physical units according to proper fibre and signal parameters^8^. For instance, for a SMF-28 fibre, $\beta _2=-20\,ps^2/km$, $\gamma =2\,^{-1} km^{-1}$ at 1.55  μμm and a pulse $q(t)=A\text {sech}(t/T0)$ with 100 ps pulse duration ($T_0=100\,ps$), the $L_{2\pi }$ length corresponds to approximately 143*km*. Note that the propagation is assumed to be lossless, i.e. a Raman amplified is used to compensate the loss. The model should not be applied to the cases with point amplification where erbium doped fibre amplifiers are used due to the continuous growth of the continuous spectrum.

**Figure 3:**
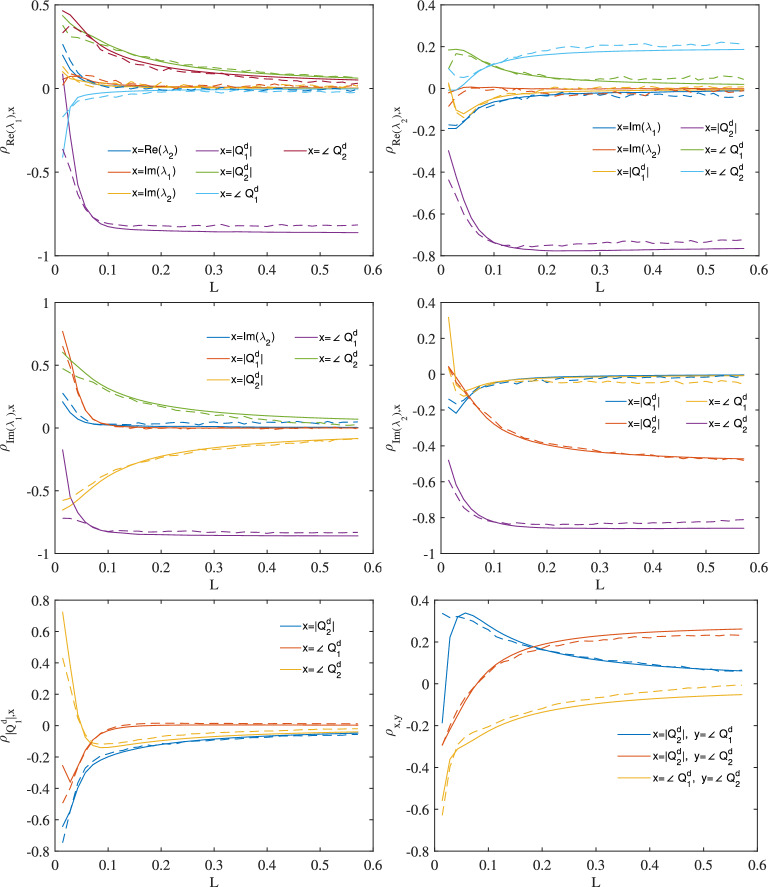
Noise linear addition approximation for the pulse with $\lambda _{1,2}=(6j,1+5j)$ and $Q^d_{1,2}=(-6j,-2j)$. Solid curves: model predictions, dashed curves: pulse propagation simulations.

Another example, Fig. 3 shows a case where the real part of the second eigenvalue is non-zero ($\lambda _{1,2}=(6j,1+5j)$). In this case, the signal separates apart into two pulses as the it propagates through the fibre. The results in Fig. 3 show good approximated predictions can still be made using the model.

### Parameter space

So far, we have described a model which allows us to find the propagation of correlations among eigenvalues, spectral magnitudes and phases at any propagation distance, by using pre-calculated covariance matrices at the beginning of the fibre. Those covariance matrices have eight degrees of freedom (two for each eigenvalue and spectrum). However, it is important to note the following properties of NFT^8^, due to which the number of degrees of freedom can be reduced.

➀: $$\frac{1}{|a|}q(t/a) \Leftrightarrow (Q^d_k,a\lambda _k,\,k=1,2)$$
➁: $$q(t-t_0) \Leftrightarrow (e^{-2j\lambda _k t_0}Q^d_k,\lambda _k,\,k=1,2)$$
➂: $$q(t)e^{j\phi } \Leftrightarrow (Q^d_k e^{-j\phi },\lambda _k,\,k=1,2)$$
➃: $$q(t)e^{-2j\omega t} \Leftrightarrow (Q^d_k,\lambda _k-\omega ,\,k=1,2)$$

Suppose $q^1(t)$ is obtained from *q*(*t*) through one of the above four transformations. It turns out that the covariance matrix for $q^1(t)$ can also be obtained directly from the covariance matrix for *q*(*t*) as well. To illustrate the idea, suppose that $q^1(t)=q(t-t_o)$. Consider the ensemble of random signals $q(t) + n(t)$ where *n*(*t*) is the additive white Gaussian noise added to *q*(*t*). Due to that *n*(*t*) is stochastic, the discrete eigenvalues and the spectral magnitudes of $q(t)+n(t)$ (denoted by $({\widetilde{Q}}_k^d,{\widetilde{\lambda }}_k,\,k=1,2)$) will be stochastic as well. Using the transformation properties, the discrete eigenvalues and the spectral magnitudes of $q(t-t_o) + n(t-t_o)$ will be distributed as $(e^{-2j{\widetilde{\lambda }}_k t_0}{\widetilde{Q}}_k^d,{\widetilde{\lambda }}_k,\,k=1,2)$. As the white noise is “time-shift” invariant in the sense that stochastic properties of *n*(*t*) and $n(t-t_o)$ are equivalent. The discrete eigenvalues and the spectral magnitudes of $q(t-t_o)+n(t)$ will also be distributed as $(e^{-2j{\widetilde{\lambda }}_k t_0}{\widetilde{Q}}_k^d,{\widetilde{\lambda }}_k,\,k=1,2)$. For that reason, we can determine the covariance matrix for $q(t-t_o)+n(t)$ from that for $q(t)+n(t)$.

A pulse with two discrete eigenvalues has 8 degrees of freedom. They are $Q^d_1$, $Q^d_2$, $\lambda _1$ and $\lambda _2$ (whereas each complex number has two degrees of freedom). However, by invoking properties ➀-➃, one can “transform” it into another two-eigenvalue pulse ${\hat{q}}(t)\Leftrightarrow ({\hat{Q}}_k^d,{\hat{\lambda }}_k,\,k=1,2)$ such that

(*i*): ${\hat{Q}}_2^d$ is a constant (say equal to ${\widetilde{Q}}_2$),
(*ii*): ${\hat{\lambda }}_2$ is purely imaginary,
(*iii*): $Im(\hat{\lambda _1}-\hat{\lambda _2})$ is a fixed constant (say denoted by $\Delta \lambda =j$).

To illustrate the construction, consider a two-eigenvalue pulse $q(t) \Leftrightarrow (Q_1^d,\lambda _1,Q_2^d,\lambda _2)$ where $\lambda _1=x_1+jy_1$ and $\lambda _2=x_2+jy_2$. For simplicity, one can assume $x_1=x_2\triangleq \omega$ and $\Delta \lambda =j$. Invoking the properties ➀ and➃, one has

25 $$\begin{aligned} q_1(t)&\triangleq q(t)e^{-2j\omega t} \Leftrightarrow (Q_1^d,jy_1,Q_2^d,jy_2) \nonumber \\ q_2(t)&\triangleq \frac{1}{|a|}q_1(t/a) \Leftrightarrow (Q_1^d,jay_1,Q_2^d,jay_2) \end{aligned}$$

where *a* can be chosen such that $ja(y_2-y_1)=\Delta \lambda$.

Next, let $t_0={(\text {ln}|\widetilde{Q_2}|-\text {ln}|Q^d_2|})/{(2a y_2 )}$. Then by ➁

$$\begin{aligned} q_3(t) \triangleq q_2(t-t_o) \Leftrightarrow \left( Q^d_1\left( \frac{|\widetilde{Q_2}|}{|Q_2^d|}\right) ^{y_1/y_2},jay_1, \frac{|\widetilde{Q_2}|Q_2^d}{|Q_2^d|},jay_2\right) \end{aligned}$$

Finally, let $\phi =\angle {Q_2^d}-\angle \widetilde{Q_2}$ such that $e^{-j\phi }\frac{|\widetilde{Q_2}|Q_2^d}{|Q_2^d|}=\widetilde{Q_2}$. By ➂

$$\begin{aligned} q_4(t) \triangleq q_3(t)e^{j\phi } \Leftrightarrow \left( e^{-j\phi }Q^d_1 \left( \frac{|\widetilde{Q_2}|}{|Q_2^d|}\right) ^{y_1/y_2},jay_1,\widetilde{Q_2},jay_2 \right) . \end{aligned}$$

For convenience, one can choose ${\widetilde{Q}}_2=-2j$ and $\Delta \lambda =j$ such that the well known two-eigenvalue pulse $2\text {sech}(t)$ satisfies the three criteria even without any transformation.

Following the discussion above, it can be concluded that, when one needs to map out the covariance matrices of the whole parameter space while designing a two-eigenvalue system, only four degrees of freedom need to be considered. They are $\lambda _1$, $|Q^d_1|/|Q^d_2|$ and the NFT phase difference $\Phi =\angle Q^d_1-\angle Q^d_2$ ($\lambda _2$ as the fourth degree if the frequency is different). Once the covariance matrices along these degrees of freedom have been obtained, the covariance matrices along all other degrees of freedom can then be computed through the transformation mentioned above.

### Numerical settings

This work relies heavily on numerical simulations. The time signal of the soliton pulses for the given $Q^d_k$ and $\lambda _k$ are calculated using the Darboux transformation method^21^ with a time window of $[-10,10]$ and a resolution of $2^{14}$ points across the time window. The time window surround the pulse is chosen such that pulse magnitudes at the edges of the time windows are smaller than -70 dB with respect to the pulse peak.

A split-step Fourier method^20^ is used for pulse propagation simulation. A step size of $10^{-5}$ is chosen for the pulse propagation simulation results in Figs. 2 and 3. In these results, White Gaussian noise where added into the simulation as stochastic noise. In Figs. 2 and 3, there are 40 points along the x-axis, each point corresponding to a segment of length $0.1L_{2\pi }$ or 1428 split steps.

To achieve high numerical accuracy, we have implemented a bi-redirection NFT algorithm^22^ using a fourth-order Runge-Kutta method. With $2^{14}$ sampling points, we can obtain accuracy more than 10 significant figures for eigenvalues and 8 significant figures for spectral magnitudes.

To obtain the covariance matrices, 5000 runs were involved in the Monte-Carlo simulation. This applies to both the pulse propagation simulation and the covariance matrix calculation at the beginning of the fibre. The Noise strength of $\kappa =0.01$ is used in all simulations.

## Conclusion

In this work, we have developed a model to estimate the covariance matrix of all 8 degrees of freedom of second-order soliton pulses at any propagation distances based on the parameters at the beginning of the fibre. The model assumes ideal Raman amplification is provided to counteract the effect of loss in the fibre. We also discussed how one can map out the covariance matrix of the whole parameter space by using only 4 degrees of freedom. The model can be used to help design communication systems such as predicting minimal distances between clusters in the constellation diagrams as well as optimising the decision boundaries for detection. Furthermore, since NFT is the same as inverse scattering transform, the model may provide useful insights into other nonlinear physics research areas such as studying ocean rouge waves^23^.
